# Neuroprotective effects of leonurine against oxygen–glucose deprivation by targeting Cx36/CaMKII in PC12 cells

**DOI:** 10.1371/journal.pone.0200705

**Published:** 2018-07-17

**Authors:** Jiao Li, Shuang Zhang, Xiaoxi Liu, Deping Han, Jianqin Xu, Yunfei Ma

**Affiliations:** College of Veterinary Medicine, China Agricultural University, Beijing, China; Fraunhofer Research Institution of Marine Biotechnology, GERMANY

## Abstract

Leonurine has been reported to play an important role in ameliorating cognitive dysfunction, inhibiting ischemic stroke, and attenuating perihematomal edema and neuroinflammation in intracerebral hemorrhage. However, the exact mechanism and potential molecular targets of this effect remain unclear. Thus, in this study we investigated the neuroprotective effects of leonurine on hypoxia ischemia injury and explored the underlying mechanisms. An *in vitro* model of oxygen–glucose deprivation (OGD)-induced PC12 cells was established to mimic ischemic-like conditions. Cell viability, apoptosis, Cx36 and pCaMKII/CaMKII expression levels were evaluated after treatment with leonurine. The Cx36-selective antagonist mefloquine and CaMKII Inhibitor KN-93 were used to investigate the neuroprotective effect of leonurine on and the involvement of Cx36/CaMKII in this process. The results revealed that cell viability decreased and cell apoptosis and the protein expression of Cx36 and pCaMKII/CaMKII increased in the OGD-induced PC12 cells. Leonurine significantly increased cell viability and decreased cell apoptosis and the protein expression of Cx36 and pCaMKII/CaMKII in the OGD-induced PC12 cells. The specific inhibitor of Cx36 and CaMKII displayed similar protective effects. Moreover, the inhibition of Cx36 reduced pCaMKII levels and the ratio of pCaMKII/CaMKII in the OGD-induced PC12 cells, and vice versa. Taken together, these results suggest that leonurine might have a protective effect on OGD-induced PC12 cells through targeting the Cx36/CaMKII pathway. Thus, leonurine appears to have potential as a preventive or therapeutic drug against ischemic-induced neuronal injury.

## Introduction

Stroke is one of the leading causes of death and disability, accounting for approximately 5.5 million deaths annually [1]. Compared with Western countries, the stroke incidence rate in China is higher, with an annual incidence of 2.5 million and an annual stroke mortality of 1.6 million [2]. Accounting for 87% of strokes [3], ischemic strokes occur when blood supply to part of the brain is decreased, resulting in glucose and oxygen deficiency and eventually brain damage [4]. Stroke-related neurologic deficits affect language, cognition, and motor functions, which severely affects patients’ quality of life [5]. Therefore, prevention and effective treatment of stroke is vital.

Thrombolytic agents, such as recombinant tissue plasminogen activator, have been the most effective therapeutic strategy for acute ischemic stroke. However, the use of thrombolytic agents is restricted to a minority of patients by the rigid 3-h time window in which they must be used [6]; moreover, they may have some negative side effects. Herbal medicine has been reported as a promising alternative choice for treating ischemic cerebral injury [7]. Therefore, greater attention should be given to natural compounds with wide therapeutic windows, clear pharmacological targets, and fewer side effects [7].

Leonurine (C_14_H_21_N_3_O_5_) is an alkaloid from *Herba leonuri*, also known as Chinese motherwort. In China, *Herba leonuri* is widely used to treat dysmenorrheal, menoxenia, and gynecological disorders. Studies have indicated that leonurine can ameliorate cognitive dysfunction by inhibiting autophagy [8], improving the antioxidant capacity of myocardium, promoting angiogenesis in ischemic myocardium, and ameliorating endothelial dysfunction caused by hyperlipidemia [9, 10]. Pretreatment with leonurine inhibits ischemic stroke [11] through antioxidant effects [12]. Leonurine can also attenuate perihematomal edema and neuroinflammation in intracerebral hemorrhage via the c-Jun N-terminal kinase pathway [13]. However, the exact mechanism and potential molecular targets underlying the protective role of leonurine in cerebral ischemia remain unclear. Connexin 36 (Cx36) is the predominant neuronal gap junction protein in the mammalian central nervous system (CNS). Research has revealed an increase in Cx36 expression following neuronal injury such as cerebral ischemia, traumatic brain injury, and epilepsy [14]. However, the exact contribution of Cx36 in cerebral ischemia remains controversial because the connexin channel family may furnish cell death as well as cell survival signals [15]. Ca^2+^/calmodulin-dependent protein kinase II (CaMKII) plays a critical role in the activity-dependent plasticity of glutamatergic synapses. CaMKII can bind to and phosphorylate Cx36 in the inferior olive neurons and synapses of mice [16], which might be an important mechanism in Cx36-promoted neuronal death.

To investigate the effects of leonurine on hypoxia ischemia injury and explore the underlying mechanisms, we established an *in vitro* model of oxygen–glucose deprivation (OGD)-induced PC12 cells to mimic ischemic-like conditions. Cell viability, apoptosis, and protein expression of Cx36 and pCaMKII/CaMKII in OGD-induced PC12 cells were evaluated after treatment with leonurine. The involvement of Cx36 and CaMKII in an OGD condition was further investigated using specific blockers.

## Materials and methods

### Cells and reagent

Differentiated rat pheochromocytoma (PC12) cells were purchased from Boster Biological Technology Co. Ltd (Wuhan, China) and preserved for reseeding and experiments. The cells were cultured in Dulbecco’s modified Eagle’s medium (DMEM) (Gibco, USA) supplemented with 10% fetal bovine serum and 100 U/mL antibiotics (penicillin and streptomycin) at 37°C in a humidified incubator containing 5% CO_2_, unless otherwise described. The culture medium was refreshed every 2 days.

Leonurine was purchased from Chengdu Pusi Biotech Co. Ltd. (Chengdu, China), and its purity was over 98.5%.

### Investigating the neuron-like characteristics of PC12 cells and localizing Cx36 and CaMKII

The PC12 cells used in this study were differentiated using nerve growth factor at the vendor’s laboratory. Immunofluorescence staining of microtubule-associated protein 2 (MAP2), a neuron-specific cytoskeletal protein, was performed to confirm the general neuron-like characteristics of the PC12 cells. The PC12 cells were seeded on coverslips and cultured for 36 h, after which they were rinsed with phosphate-buffered saline (PBS) three times. After fixation in 4% paraformaldehyde for 15 min and permeabilization with 0.2% Triton X-100 at 0°C, the cells were blocked with 1% bovine serum albumin for 30 min. Subsequently, the cells were incubated with the mouse antibody to MAP2 (1:100; BM1243, Boster Co. Ltd., China) overnight at 4°C, followed by incubation with biotinylated donkey antibody to mouse IgG (1:100, 715-065-151, Jackson, USA) and then with AlexaFluor594-conjugated streptavidin (1:100; Invitrogen, USA). The nuclei were counterstained with 4’,6’-diamidino-2-phenylindole (DAPI; Roche, USA). Images were captured using a fluorescence microscope (Nikon, Japan).

To determine the localization of Cx36 and CaMKII in the PC12 cells, double immunofluorescence staining was performed. The cells were incubated overnight with the primary antibodies including rabbit antibody to Cx36 (1:100, 516300, Invitrogen) and mouse antibody to CaMKII (1:500, ab22609, Abcam, USA), followed by incubation with the secondary antibodies including horseradish peroxidase (HRP)-goat-anti-rabbit-IgG (1:100, K008, Kemei Bo Rui Technology, China) and biotinylated donkey antibody to mouse IgG (1:100, 715-065-151, Jackson). The cells were then incubated with tyramide signal amplification plus 2,4-dinitrophenyl (TSA–DNP; 481106, Amplification Reagent, USA) for 20 min, followed by incubation with AlexaFluor594-conjugated streptavidin and AlexaFluor488-conjugated DNP (1:100; Scientific Research Special, Japan) for 30 min. DAPI was used for nuclei staining, and images were captured using a confocal microscope (Olympus, Japan).

### Establishing a model of OGD-induced PC12 cells

To mimic ischemic-like conditions *in vitro*, a model of OGD-induced PC12 cells was established with reference to previous studies [17, 18]. Specifically, the PC12 cells were incubated in glucose-free DMEM, which was supplemented with 10% fetal bovine serum and 100 U/mL antibiotics (penicillin and streptomycin), in a hypoxia chamber (Thermo Scientific, USA) containing 1% O_2_, 94% N_2_, and 5% CO_2_. After incubation for a short period, the cells were moved to normal culturing conditions (glucose-free DMEM was replaced with DMEM containing 4500 mg/L of glucose) and cultured for 24 h for use in further experiments.

### Evaluation of the cell morphology, viability, and apoptosis of OGD-induced PC12 cells

To evaluate cell morphology, viability, and apoptosis and to determine the appropriate length of OGD induction, the PC12 cells were cultured under OGD for 0, 1, 2, 4, 6, 8, 10, or 12 h, and they were then cultured under normal conditions for 24 h as previously described. Normal controls were appropriately set, and cell morphology was observed under a microscope (Nikon, Japan).

Cell viability was quantified using cell counting kit-8 (Dojindo, Japan). The PC12 cells were seeded at 100 μL/well in a 96-well plate according to the manufacturer’s instructions. After OGD induction, 10 μL of CCK-8 solution was added to each well and incubated for 1 h. The relative cell viability was determined by measuring absorbance at 450 nm using an iMark™ Microplate Absorbance Reader (BIO-RAD, USA).

Cell apoptosis was detected through dual acridine orange/ethidium bromide (AO/EB) staining. The PC12 cells were cultured on glass coverslips as previously described and then washed with PBS. A mixture of 100 μg/mL AO and 100 μg/mL EB (AO/EB, Sigma, St. Louis, MO, USA) was added to each coverslip, and each coverslip was covered on a slide and kept in a dark place for 15 min. The morphology of the apoptotic cells was observed under a fluorescent microscope (Nikon, Japan) according to the manufacturer’s instructions.

According to the changes in PC12 cell morphology, apoptosis, and viability, the appropriate OGD induction time was chosen for the subsequent experiments.

### Evaluation of Cx36 and pCaMKII/CaMKII expression in OGD-induced PC12 cells

The changes in Cx36 and pCaMKII/CaMKII expression in the OGD-induced PC12 cells were investigated. The PC12 cells were cultured under OGD for 2 h, followed by normal culture for 1, 2, 4, 6, 12, or 24 h. Proteins of the PC12 cells were extracted using a whole-cell lysis assay kit (KeyGEN BioTECH, China). The cells were lysed in lysis buffer supplemented with 1× protease inhibitor cocktail. Protein was quantified using a bicinchoninic acid protein assay kit (CWBIO, China). Equivalent amounts of proteins were separated using sodium dodecyl sulphate-polyacrylamide gel electrophoresis (8%–10%), transferred onto a polyvinylidene difluoride membrane, and incubated with primary antibodies including rabbit anti-Cx36 (1:500; 516300, Invitrogen, Japan), rabbit anti-pCaMKII (1:500; ab52476, Abcam, USA), rabbit anti-CaMKII (1:1000; 3356, Cell Signaling Technology, USA), and mouse anti-β-actin (1:5000; 50201, Kemei Bo Rui, China). They were then incubated with HRP-conjugated secondary antibodies including HRP-goat-anti-rabbit-IgG (1:5000; K008, Kemei Bo Rui Technology, China) and HRP-goat-anti-mouse-IgG (1:5000, E030110, EarthOx, USA). The protein expression levels of Cx36 and pCaMKII/CaMKII were quantified using a Tanon 5200 chemiluminescence system (Tanon, China).

### Evaluating the effect of leonurine, mefloquine, and KN93 on OGD-induced PC12 cells

To analyze the neuroprotective effect of leonurine on and the involvement of Cx36/CaMKII in OGD-induced PC12 cells, the PC12 cells were incubated under OGD for 2 h and then separately incubated with the following reagents at different doses for 3 h: the Cx36-selective antagonist mefloquine (MFQ) (1.25 μL as low dose, 2.5 μM as middle dose, and 5 μM as high dose), CaMKII Inhibitor KN-93 (0.5 μM as low dose, 1 μM as middle dose, and 2μM as high dose), and leonurine (50 μg/mL as low dose, 100 μg/mL as middle dose, and 200 μg/mL as high dose). The cells were then cultured under normal conditions for 24 h.

The cells were collected after 24 h. Cell viability and Cx36 and pCaMKII/CaMKII expression levels were investigated as previously described. Cell apoptosis was evaluated using an Annexin V-FITC/PI apoptosis detection kit (KeyGen BioTech Co. Ltd., Nanjing, China). After trypsinization, single-cell suspensions were extracted and washed with PBS. The cells were resuspended in 500 μL of binding buffer and stained with Annexin V-FITC and PI for 15 min according to the manufacturer’s instructions. The samples were then analyzed using a flow cytometer (BD, Franklin Lakes, NJ, USA) with a maximal excitation wavelength at 488 nm and emission at 530 nm.

### Statistical analysis

Statistical analyses were performed using SPSS 17.0 (SPSS, Inc. an IBM Company, Chicago, IL, USA). All data are presented as mean ± standard deviation (SD) or percentage. Comparisons between two groups were performed using a paired t-test. Comparisons between three or more groups were performed using one-way analysis of variance. P < 0.05 indicated statistical significance.

## Results

### Neuron-like characteristics of PC12 cells and colocalization of Cx36 and CaMKII

**Fig 1:**
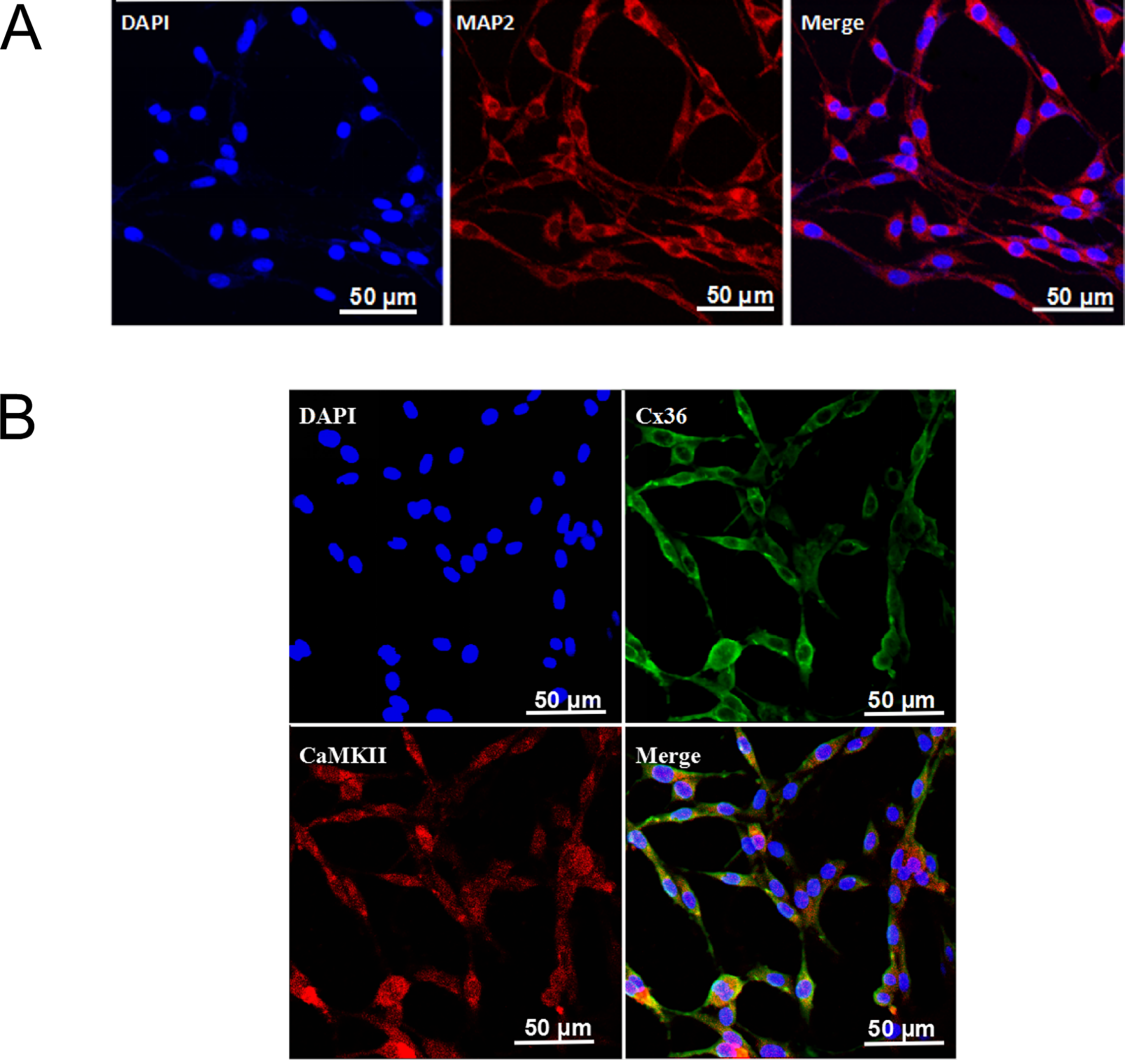
Immunofluorescence staining of MAP2 in PC12 cells and colocalization of Cx36 and CaMKII in PC12 cells. (A) PC12 cells exhibited neuron-like characteristics. MAP2-positive staining was in red, and the nuclei were stained with DAPI in blue. (B) Colocalization of Cx36 and CaMKII in PC12 cells. The positive Cx36 staining was in green and CaMKII was in red. The nuclei were in blue.

Almost all of the PC12 cells exhibited the basic structural features of neurons. Positive MAP2 staining was observed in the cell membrane and neurites. The neurite lengths were approximately 1.5–2 times the cell body (Fig 1A). Spatial colocalization of positive Cx36 and CaMKII staining in the PC12 cells was observed (Fig 1B), indicating a spatial and structural basis for the interaction between Cx36 and CaMKII.

### Cell morphology, viability, and apoptosis of OGD-induced PC12 cells

**Fig 2:**
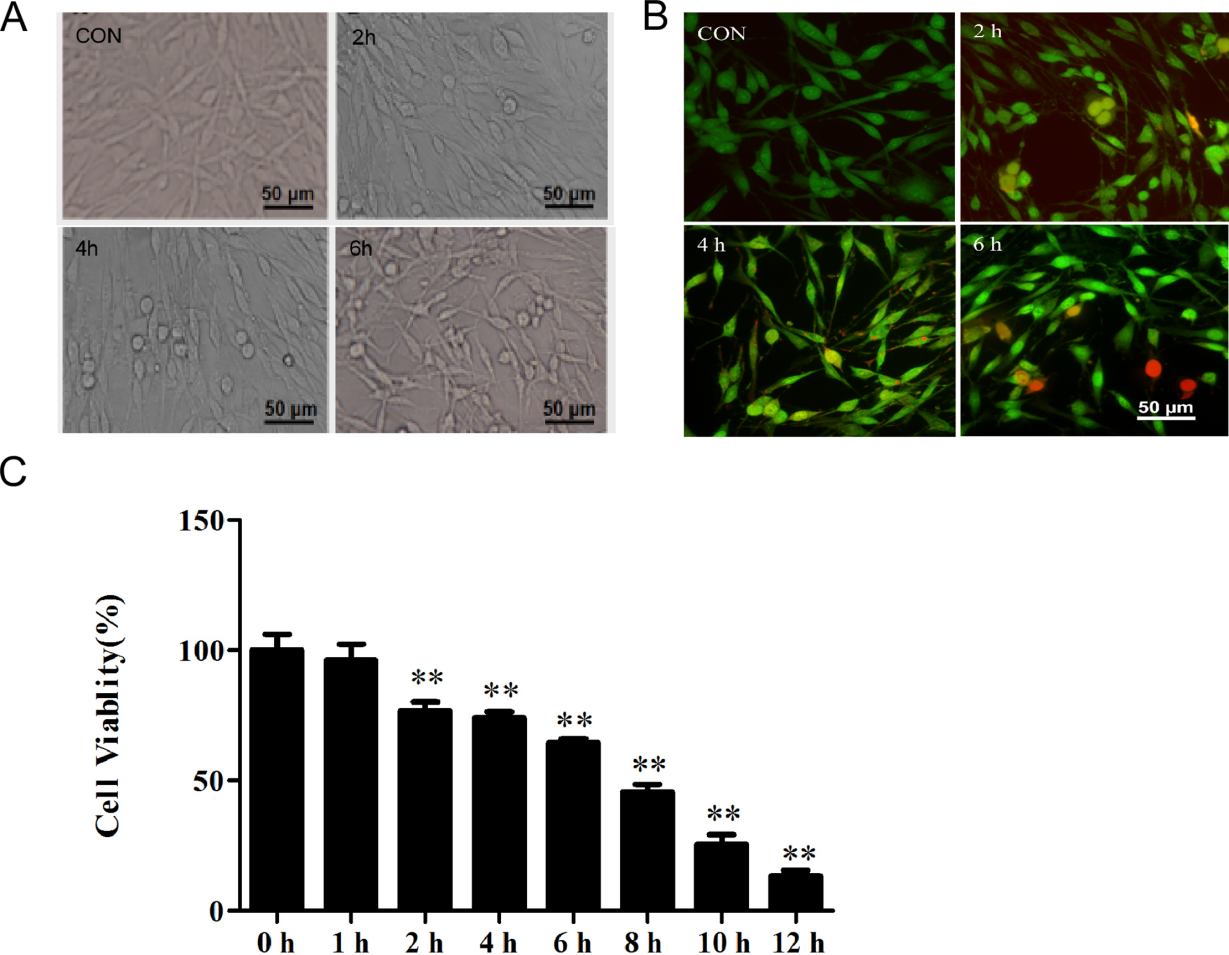
Effects of OGD on the morphology, cell apoptosis, and viability of PC12 cells. PC12 cells were cultured under OGD for 0, 1, 2, 4, 6, 8, 10, or 12 h and then cultured under normal conditions for 24 h. Cell morphology, apoptosis, and viability were evaluated. (A) PC12 cell morphology. OGD-induced PC12 cells exhibited round, slender, and degenerated morphologies. (B) Cell apoptosis. In the 2-h-OGD-induced group, early apoptotic cells emitted yellow–green fluorescence. In the 6-h-OGD-induced group, late apoptotic cells with concentrated, localized orange and red nuclear staining were detected. (C) Cell viability decreased with the length of OGD induction. CON, control group. ^*^P < 0.05 compared with the control (0 h) group. ^**^P < 0.01 compared with the control (0 h) group.

Cell morphology, apoptosis, and viability were investigated in the OGD-induced PC12 cells. The OGD-induced PC12 cells exhibited round, slender, and degenerated morphologies (Fig 2A). In addition, cell viability was lower in the OGD-induced cells compared with the control group (100.10% ± 2.49%), and it decreased with the length of OGD induction (Fig 2C). No significant apoptosis was observed in the control group. In the 2-h-OGD-induced group, early apoptotic cells with yellow–green fluorescence were detected. As the length of OGD induction increased, the number of early apoptotic cells increased; moreover, late apoptotic cells with concentrated, localized orange and red nuclear EB staining were detected in the 6-h-OGD-induced group (Fig 2B).

These results indicate that PC12 cell morphology, apoptosis, and cell viability significantly changed after 2 h of OGD induction. Therefore, the 2-h-OGD-induced PC12 cells were used in the subsequent experiments.

### Protein expression of Cx36 and pCaMKII/CaMKII in OGD-induced PC12 cells

**Fig 3:**
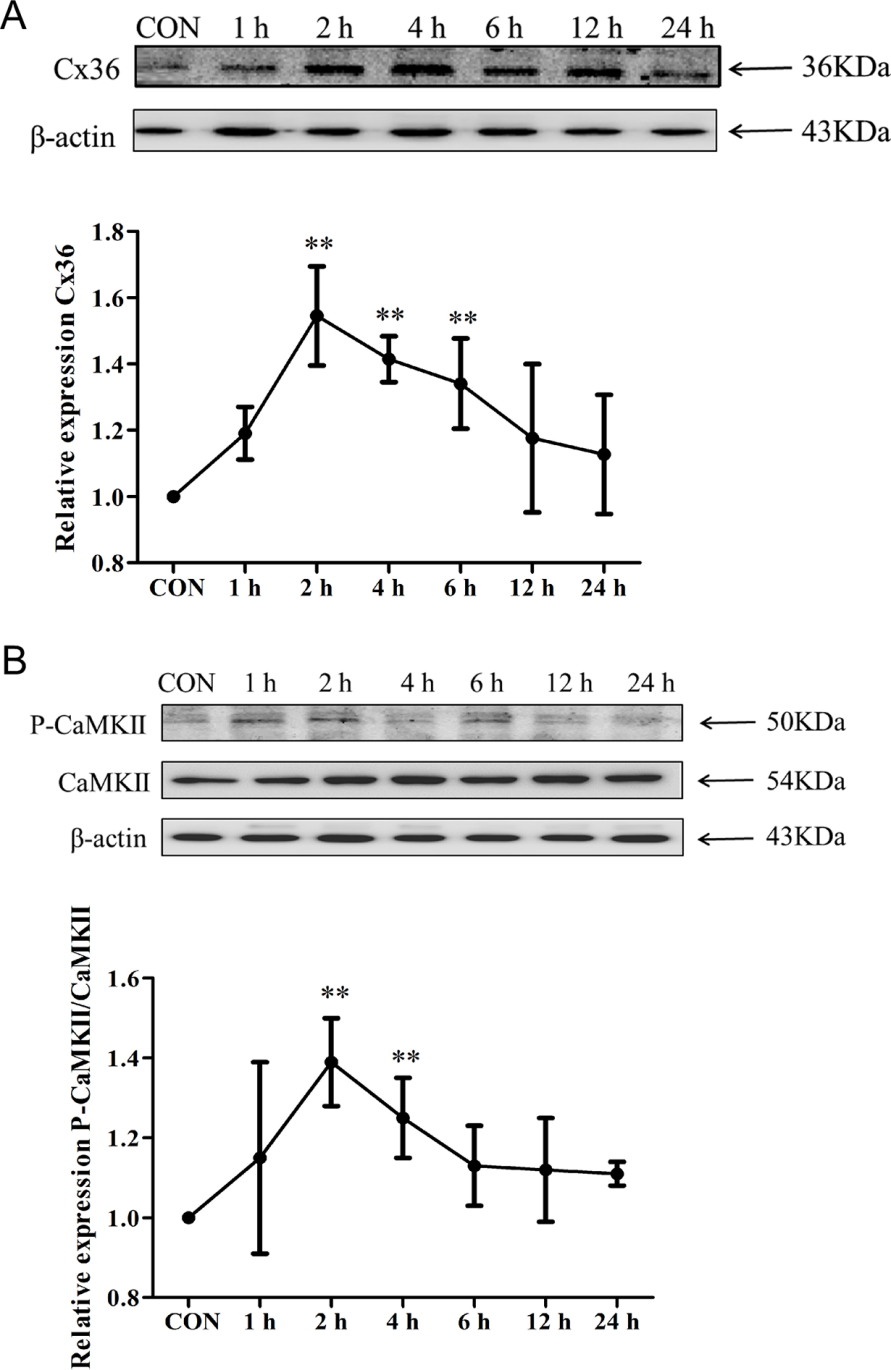
Protein expression of Cx36 and pCaMKII/CaMKII in OGD-induced PC12 cells. PC12 cells were incubated under OGD for 2 h and then cultured under normal conditions for 1, 2, 4, 6, 12, and 24 h. Protein expression levels of Cx36 and pCaMKII/CaMKII were evaluated through double immunofluorescence staining. (A) Cx36 protein expression. (B) Protein expression levels of CaMKII and pCaMKII. CON, control group. ^**^P < 0.01 compared with the control (0 h) group.

After the 2-h-OGD-induced PC12 cells were cultured under normal conditions for 1 h, the protein expression of Cx36 increased by 19% compared with the control group (P > 0.05); it increased by 55% after the cells were cultured under normal conditions for 2 h and then gradually declined with culturing time but remained higher than the control group (Fig 3A). The changes in the pCaMKII/CaMKII ratio were similar to Cx36 (Fig 3B). These results suggest that Cx36 and pCaMKII/CaMKII might be involved in the OGD conditioning of PC12 cells.

### Protective effects of leonurine on OGD-induced PC12 cells and the involvement of Cx36 and pCaMKII/CaMKII

To analyze the neuroprotective effect of leonurine on and the involvement of Cx36/CaMKII in OGD-induced PC12 cells, the cell viability, apoptosis, and protein expression of Cx36 and pCaMKII/CaMKII were evaluated after the 2-h-OGD-induced PC12 cells were separately incubated with leonurine, MFQ, and KN-93.

**Fig 4:**
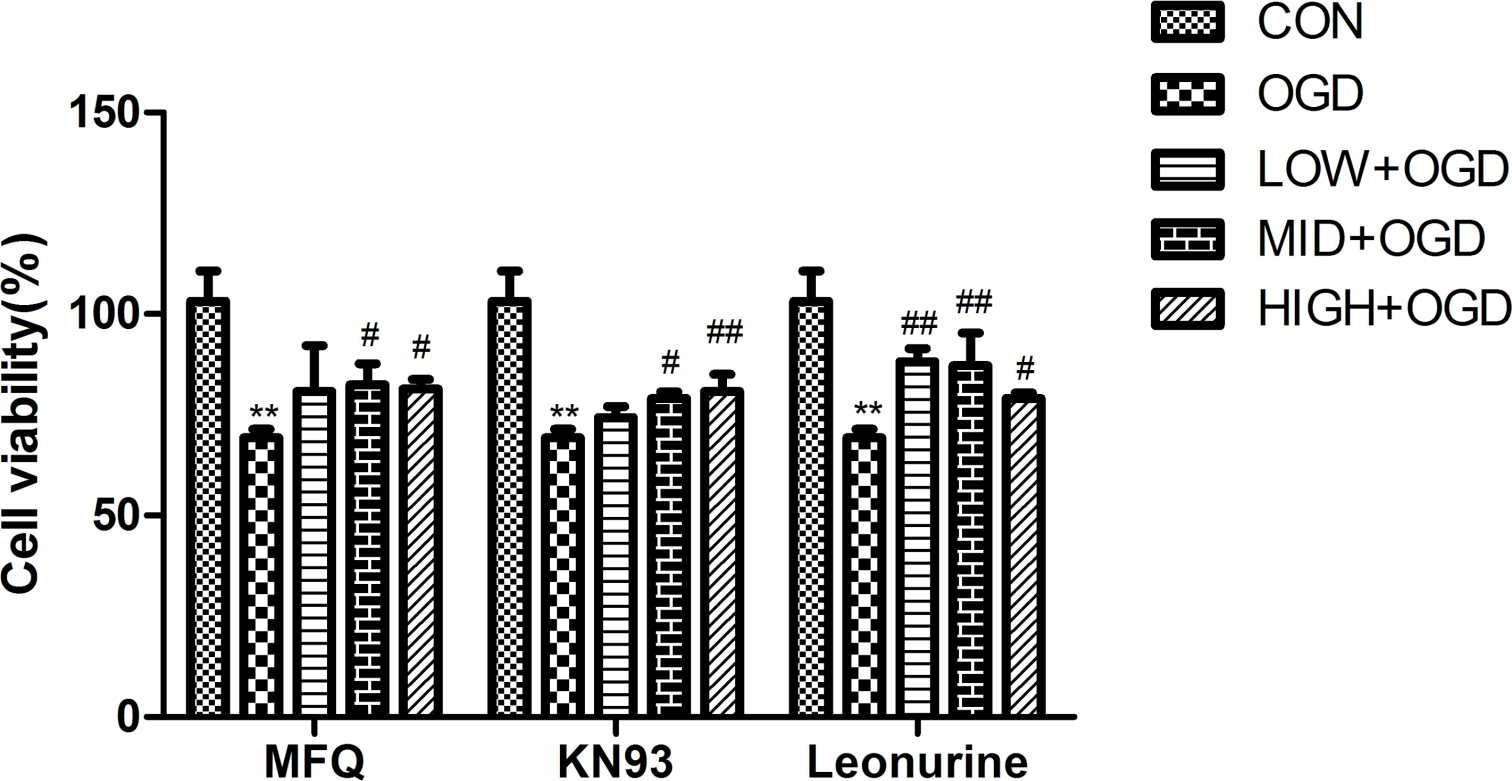
Effect of Leonurine, MFQ, and KN93 on the cell viability of PC12 cells. PC12 cells were incubated under OGD for 2 h, and they were then separately incubated with the following reagents for 3 h: MFQ (5 μM), KN-93 (2 μM), and leonurine (50, 100, and 200 μg/mL). Then the cells were cultured under normal conditions for 24 h. Cell viability was assessed using cell counting kit-8. CON, normal control group; OGD, OGD-for-control group; LOW+OGD, low-dose leonurine/MFQ/KN93 groups; MID+OGD, middle-dose leonurine/MFQ/KN93 groups; HIGH+OGD, high-dose leonurine/MFQ/KN93 groups. ^*^P < 0.05 compared with the normal control (0-h OGD) group. ^**^P < 0.01 compared with the normal control (0-h OGD) group. ^#^*P* < 0.05 compared with the OGD-for-control group. ^##^*P* < 0.01 compared with the OGD-for-control group.

Compared with the OGD-for-control group, cell viability was significantly increased by 13.15% and 12.14% in the middle-dose MFQ group (P < 0.05) and high-dose MFQ group (P < 0.05), respectively. Cell viability was also significantly increased by 9.74% and 11.49% in the middle-dose KN-93 group (P < 0.05) and high-dose KN-93 group (P < 0.01). Cell viability was significantly increased by 18.93%, 17.90%, and 9.74% in the low-dose leonurine group (P < 0.01), middle-dose leonurine group (P < 0.01), and high-dose leonurine group (P < 0.01), respectively, compared with the OGD-for-control group (Fig 4).

**Fig 5:**
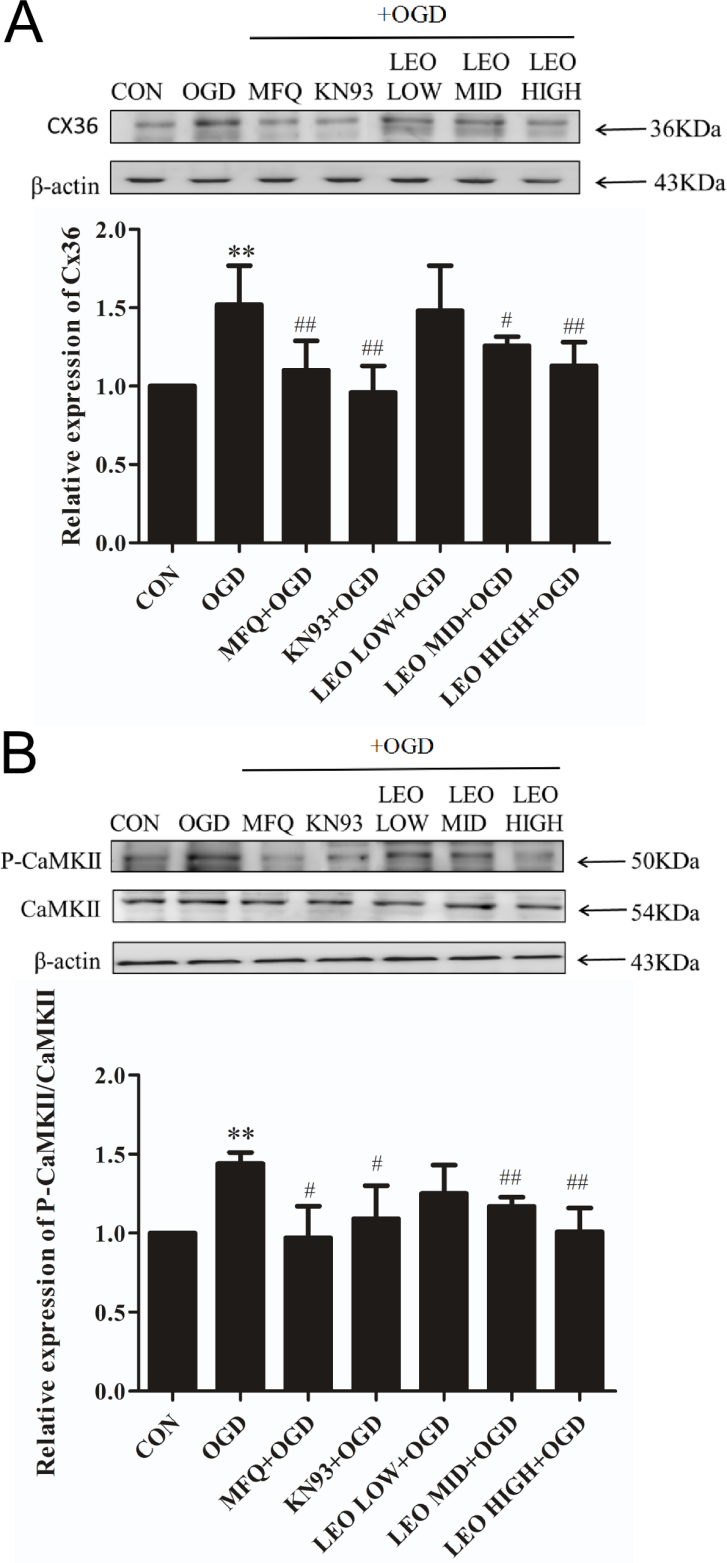
Effect of leonurine, MFQ, and KN93 on protein expression of Cx36 and pCaMKII/CaMKII in OGD-induced PC12 cells. PC12 cells were incubated under OGD for 2 h and then separately incubated with the following reagents for 3 h: MFQ (5 μM), KN-93 (2 μM), and leonurine (50, 100, and 200 μg/mL). Then, the cells were cultured under normal conditions for 24 h. Protein expression was evaluated through Western blot analysis. (A) Protein expression of Cx36. (B) Protein expression of CaMKII and pCaMKII. CON, normal control group; OGD, OGD-for-control group; MFQ+OGD, MFQ-treated group; KN93+OGD, KN93-treated group; LEO LOW+OGD, low-dose leonurine group; LEO MID+OGD, middle-dose leonurine group; LEO HIGH+OGD, high-dose leonurine group. ^*^P < 0.05 compared with the normal control (0 h OGD) group. ^**^P < 0.01 compared with the normal control (0-h OGD) group. ^#^*P* < 0.05 compared with the OGD-for-control group. ^##^*P* < 0.01 compared with the OGD-for-control group.

Cx36 expression was significantly higher in the OGD-for-control group than in the normal control group (P < 0.001). Compared with the OGD-for-control group, Cx36 expression in the middle-dose and high-dose leonurine groups significantly decreased by 24.34% and 27.63%, respectively (P < 0.05). MFQ and KN-93 treatment also decreased the protein expression of Cx36 in the OGD-induced PC12 cells (P < 0.05; Fig 5A).

The ratio of pCaMKII/CaMKII significantly increased by 44% in the OGD-for-control group compared with the normal control group (P < 0.05). The middle-dose and high-dose leonurine treatments decreased the ratio of pCaMKII/CaMKII by 13.19% and 25.00% compared with the OGD-for-control group (P < 0.05). The middle-dose and high-dose MFQ treatments also significantly decreased the ratio of pCaMKII/CaMKII by 22.92% and 34.85%, respectively, compared with the OGD-for-control group (P < 0.05). The ratios of pCaMKII/CaMKII in the middle-dose and high-dose KN-93 groups were lower (P < 0.05) than those in the OGD-for-control group (Fig 5B).

**Fig 6:**
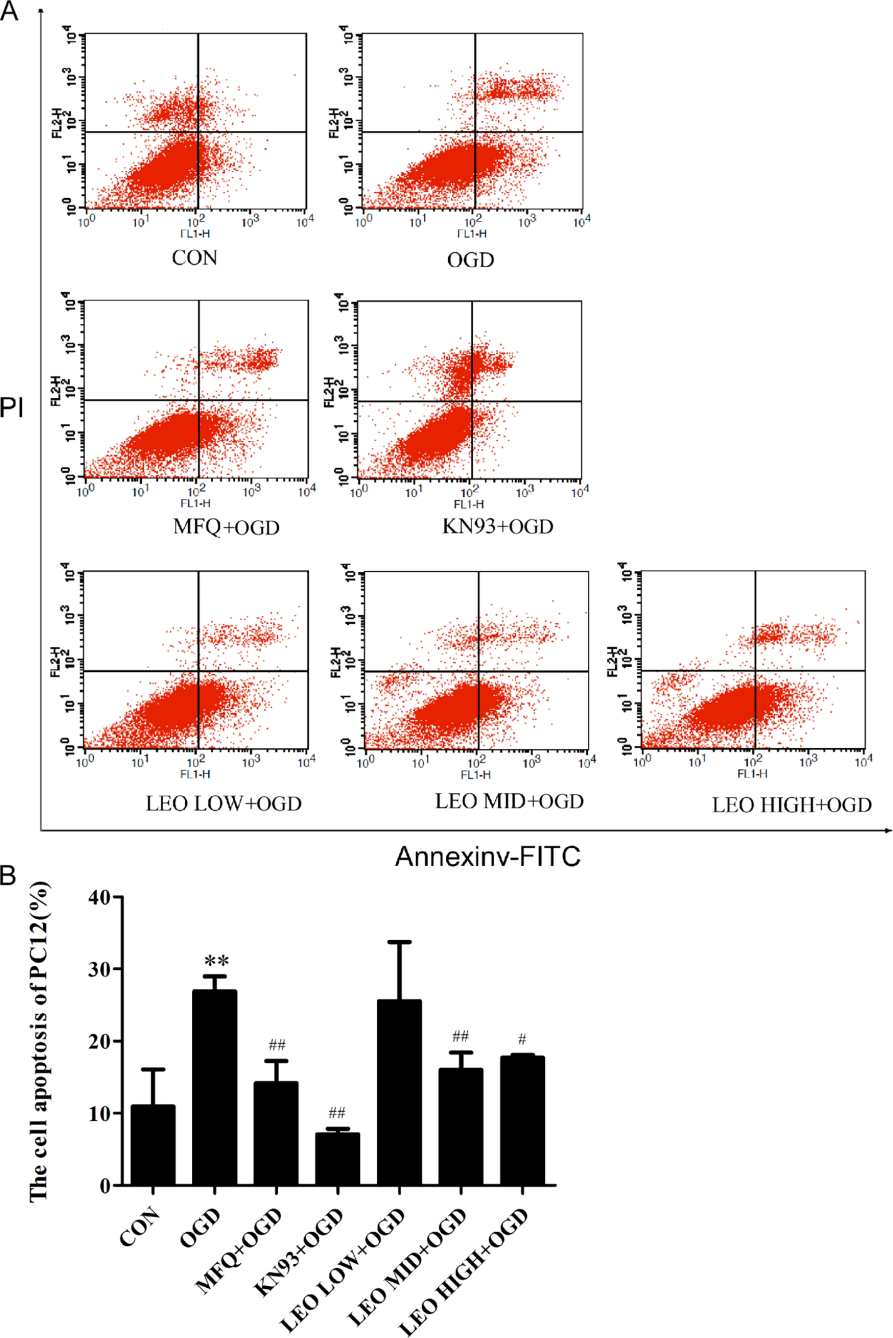
Effect of Leonurine, MFQ,and KN93 on cell apoptosis in OGD-induced PC12 cells. PC12 cells were incubated under OGD for 2 h, and they were then separately incubated with MFQ (5 μM), KN-93 (2 μM), and leonurine (50, 100, and 200 μg/mL, respectively) for 3 h, followed by culturing under normal conditions for 24 h. Cell apoptosis was detected using Annexin V-FITC/PI staining. (A) Results of flow cytometry. (B) Quantification of cell apoptosis. CON, normal control group; OGD, OGD-for-control group; MFQ+OGD, MFQ-treated group; KN93+OGD, KN93-treated group; LEO LOW+OGD, low-dose leonurine group; LEO MID+OGD, middle-dose leonurine group; LEO HIGH+OGD, high-dose leonurine group. ^*^P < 0.05 compared with the normal control group. ^**^P < 0.01 compared with the normal control group. ^#^*P* < 0.05 compared with the OGD-for-control group. ^##^*P* < 0.01 compared with the OGD-for-control group.

Apoptosis of the PC12 cells treated with leonurine, MFQ, and KN-93 was quantified through flow cytometry. The apoptotic rate in the OGD-for-control group was 26.87%, significantly higher than that in the normal control group (P < 0.01). Compared with the OGD-for-control group, the cell apoptotic rates in the middle-dose leonurine group, high-dose leonurine group, and high-dose MFQ group were significantly lower (P < 0.01). The apoptotic rate in the 2-μM-KN93-treated group was 7.09%, significantly lower than that in the OGD-for-control group (P < 0.01). However, the necrosis rate was 11.32%, which was significantly higher (P < 0.05) than that in the control group (2.10%; Fig 6A and 6B).

## Discussion

Leonurine has been reported to have cardioprotective effects against ischemia-induced myocardial injury by reducing intracellular reactive oxygen species levels and increasing antiapoptosis-associated protein expression [19]. In addition, some studies have reported that leonurine can reduce the infarction area of the cerebral cortex and repair neurological damage [12, 20, 21]. In this study, we found that leonurine had neuroprotective effects on OGD-induced PC12 cells through downregulating the protein expression of Cx36 and pCaMKII/CaMKII.

To mimic ischemic-like conditions *in vitro*, we induced PC12 cells with OGD. The results reveal that leonurine significantly improved cell viability and reduced cell apoptosis without obvious cytotoxicity in the OGD-induced PC12 cells. This indicates that leonurine might play a neuroprotective role in PC12 cells under OGD conditions. Moreover, Cx36 expression and the ratio of pCaMKII/CaMKII increased in the OGD-induced PC12 cells. After leonurine treatment, CaMKII phosphorylation, Cx36 expression, and the ratio of pCaMKII/CaMKII were all significantly decreased. This suggests that both proteins were involved in the neuroprotective mechanism.

Cx36 is the predominant neuronal gap junction protein in the mammalian CNS [22]. Gap junctions are specialized cell–cell junctions that directly link the cytoplasm of neighboring cells and provide cytoplasmic continuity between adjacent cells; thus, small molecules such as cyclic adenosine monophosphate, inositol trisphosphate, and Ca^2+^ can be transferred across cells [23]. The exact contribution of the gap junction in cerebral ischemia remains controversial because the connexin channel family may furnish cell death as well as cell survival signals. Treatment with the gap junction blocker carbenoxolone was reported to differentially accelerate N-methyl-D-aspartate-induced cell death in both astrocytes and neurons [24]; the protein expression of Cx36 and the coupling of neurons by gap junctions play an important role in many developmental events [25]. Previous studies have reported an increase in Cx36 expression levels following neuronal injury such as cerebral ischemia [26, 27], traumatic brain injury [28], and epilepsy [29]. One study revealed that Cx36 immunoreactivity was induced in neurons for 1 h and then gradually decreased to normal [30]. CaMKII plays a critical role in the activity-dependent plasticity of glutamatergic synapses. CaMKII was reported to bind to and phosphorylate Cx36 in the inferior olive neurons and synapses of mice [16], which may be an important mechanism in Cx36-promoted neuronal death.

We investigated the protein expression of Cx36 and pCaMKII/CaMKII in OGD-induced PC12 cells. Our results reveal that the protein expression of Cx36 and the ratio of pCAMKII/CaMKII increased rapidly after OGD induction and then gradually decreased with time. When the OGD-induced PC12 cells were treated with a specific Cx36 blocker, MFQ, cell viability increased. This indicates that Cx36 may be a potential target to protect neurons from ischemia damage. Additionally, MFQ inhibited the phosphorylation of CaMKII and significantly decreased the ratio of pCaMKII/CaMKII in the OGD-induced PC12 cells, suggesting an interaction between Cx36 and CaMKII during ischemia-induced neuron damage. KN93 is a selective CaMKII inhibitor, and treating OGD-induced PC12 cells with KN93 revealed similar effects to the Cx36 blocker; KN93 significantly inhibited the phosphorylation of CaMKII and significantly decreased the ratio of pCaMKII/CaMKII as well as the protein expression of Cx36 in the OGD-induced PC12 cells.

Immunofluorescence staining revealed that CaMKII and Cx36 were colocalized in the PC12 cells. CaMKII was reported to effectively bind to two juxta-membrane cytoplasmic domains of phosphorylate Cx36 [16]. Moreover, homozygous Cx36 KO mice were revealed to exhibit reduced CaMKII levels in the striatum compared with heterozygous mice [31]. Therefore, we speculate that, in PC12 cells, CaMKII bound with Cx36 and the interaction between CaMKII and Cx36 might influence protein expression and/or stability. Our results also suggest that Cx36/CaMKII might be a potential therapeutic target for treating cerebral ischemia. However, a CaMKII inhibitor may not be appropriate because KN93 treatment decreased cell apoptosis and increased the proportion of necrotic cells. Thus, inhibiting CaMKII phosphorylation by KN93 may be cytotoxic for PC12 cells.

Under ischemic conditions, the lack of glucose, oxygen, ATP, and phosphocreatine [32] leads to uncontrolled membrane depolarization and rapid increase in intracellular Ca^2+^, which is a result of influx through a variety of Ca^2+^-permeable ion channels and release from intracellular stores [33]. Extracellular accumulation of glutamate and other neurotransmitters also activates receptors that lead to further depolarization and increases intracellular Ca^2+^ and Na^+^, a self-reinforcing cycle of events known as excitotoxicity. The rise in intracellular Ca^2+^ rapidly activates CaMKII through binding of Ca^2+^-calmodulin. Although CaMKII is only one of many Ca^2+^-stimulated enzymes that are activated following ischemia, CaMKII appears to be one of the major upstream regulators in postischemia neurotoxicity because its inhibition can prevent the majority of infarct formation processes [34]. The neuroprotective effect of CaMKII inhibitors was observed in this study, although a cytotoxic effect was also noted. We speculate that the inhibition of CaMKII may affect multiple Ca^2+^-dependent processes aside from cell death induction. This requires further study.

This study’s primary limitation is that the osmolarity of the medium used in this study should have been checked before, during, and after OGD exposure. However, we can still conclude that leonurine might have a protective effect on OGD-induced PC12 cells without obvious toxicities by targeting the Cx36/CaMKII pathway. Thus, leonurine may be useful as a preventive or therapeutic drug against ischemic-induced neuronal injury.
